# Vitamin D’s Impact on Cancer Incidence and Mortality: A Systematic Review

**DOI:** 10.3390/nu17142333

**Published:** 2025-07-16

**Authors:** Sunil J. Wimalawansa

**Affiliations:** CardioMetabolic & Endocrine Institute, North Brunswick, NJ, USA; suniljw@hotmail.com

**Keywords:** 25(OH)D, 1,25(OH)_2_D, epidemiology, malignancy, metastasis, micronutrients, prevention, public health, health risks, susceptibility

## Abstract

**Background/Objectives**: Adequate vitamin D levels are essential for various physiological functions, including cell growth, immune modulation, metabolic regulation, DNA repair, and overall health span. Despite its proven cost-effectiveness, widespread deficiency persists due to inadequate supplementation and limited sunlight exposure. **Methods**: This systematic review (SR) examines the relationship between vitamin D and the reduction of cancer risk and mortality, and the mechanisms involved in cancer prevention. This SR followed the PRISMA and PICOS guidelines and synthesized evidence from relevant studies. **Results**: Beyond genomic actions via calcitriol [1,25(OH)_2_D]-receptor interactions, vitamin D exerts cancer-protective effects through mitigating inflammation, autocrine, paracrine, and membrane signaling. The findings reveal a strong inverse relationship between serum 25(OH)D levels and the incidence, metastasis, and mortality of several cancer types, including colon, gastric, rectal, breast, endometrial, bladder, esophageal, gallbladder, ovarian, pancreatic, renal, vulvar cancers, and both Hodgkin’s and non-Hodgkin’s lymphomas. While 25(OH)D levels of around 20 ng/mL suffice for musculoskeletal health, maintaining levels above 40 ng/mL (100 nmol/L: range, 40–80 ng/mL) significantly lowers cancer risks and mortality. **Conclusions**: While many observational studies support vitamin D’s protective role in incidents and deaths from cancer, some recent mega-RCTs have failed to demonstrate this. The latter is primarily due to critical study design flaws, like recruiting vitamin D sufficient subjects, inadequate dosing, short durations, and biased designs in nutrient supplementation studies. Consequently, conclusions from these cannot be relied upon. Well-designed, adequately powered clinical trials using appropriate methodologies, sufficient vitamin D_3_ doses, and extended durations consistently demonstrate that proper supplementation significantly reduces cancer risk and markedly lowers cancer mortality.

## 1. Introduction

In most humans, vitamin D is typically synthesized through skin exposure to sunlight via ultraviolet B rays (UVB), particularly during summer-like conditions [1]. It is metabolized primarily in the liver and peripheral target cells to form 25(OH)D (calcifediol) [2,3]. Part of the calcifediol produced in the liver gets converted to the active form, 1,25(OH)_2_D (calcitriol), in the renal tubules. At the same time, another portion, along with D_3_, is stored in muscle and fat cells via an active mechanism [4,5]. The remainder of D_3_ and 25(OH)D in the circulation diffuse (and endocytosed) into peripheral target cells, including immune cells [6], enabling local synthesis of calcitriol [3,7]. This locally synthesized calcitriol from both D_3_ and calcifediol is crucial for their autocrine, paracrine signaling, and genomic functions [8,9,10]. Additionally, vitamin D also has non-genomic functions [11,12,13], such as membrane stabilization [12,14,15] in epithelial and immune cells [16,17].

Over half of the global population experiences vitamin D deficiency at some point during the year, surpassing iron deficiency [18,19,20,21]. This is prevalent across various regions, regardless of geographic location, due to multiple factors. Limited winter sunlight, sun avoidance (use of umbrellas, sunscreen, and clothing), high melanin content or scarring in the skin, and aging significantly reduce cutaneous vitamin D synthesis [22,23,24]. S-avoiding behavior is prevalent in tropical regions that limit vitamin D production [25]. Vitamin D synthesis drops to negligible levels during winter, early mornings, and late afternoons, also when individuals wear excessive clothing or apply heavy sunscreen [26,27,28,29].

Ironically, regions that have increasingly adopted sun-avoidant behaviors, such as Middle Eastern countries (to avoid harsh climatic conditions) and India (particularly among women due to concerns about skin darkening) over the past four decades, have reported increased prevalence of hypovitaminosis D [30]. Overall, lower serum 25(OH)D levels are associated with increased incidences of cancer [31,32], infections, autoimmune diseases [33], and chronic conditions [20,34,35]. Most epidemiological and observational studies emphasize the role of adequate vitamin D in reducing cancer mortality rather than incidence [28], with benefits largely attributed to solar UVB exposure [36]. Mortality is a more distinctly defined endpoint than symptoms, which provide more substantial beneficial results. However, cancer outcomes vary significantly depending on the timing of diagnosis, screening, and the interventions used. The variability observed across studies is largely attributable to differences in study populations and the stage of cancer at the time of recruitment.

### 1.1. Systemic Challenges and Clinical Trial Designs Using Vitamin D

While case-control, prospective, and cohort studies support a stronger association between hypovitaminosis and cancer [6,37], recent randomized controlled trials (RCTs) assessing vitamin D supplementation and cancer incidence have reported less convincing evidence [38,39,40,41,42]. Observational studies are also susceptible to confounding bias, but are minimized with fewer manipulations (straightforward study designs without introducing biases) and have a larger sample size and longer study duration. RCT study designs are complex and inherently unsuitable for testing micronutrients [43,44].

Notably, recent large RCTs, such as the VITAL study [45,46] and others, have been criticized for significant design flaws [25,45,47], which has led to the conclusion that there are non-significant effects of vitamin D on primary outcomes, including the prevention and mortality of cancer and cardiovascular events [48,49]. Nevertheless, reductions in acute respiratory infections and cancer mortality have been reported [47]. Negative findings in these trials stem from the enrollment of participants without baseline deficiency, design bias, inadequate doses, short durations, and infrequent administration, often less than once a month [43,45,46,50].

Improvements in disease outcomes, such as in cancer, are unlikely without properly addressing study design failures. Despite this, due to a lack of understanding of the fundamental biological differences between pharmaceuticals and micronutrients, some research groups continue to use RCTs [45,51,52] and the Mendelian randomization process for vitamin D (and other micronutrient) clinical studies. For micronutrients, these two methods are inferior to ecological clinical studies for evaluating nutrients and, therefore, are not the proper methods for testing and evaluating the efficacy of micronutrients, such as vitamin D [42,43,47,53].

### 1.2. The Importance of Adjusting for Confounders in Clinical Research

To draw valid conclusions, it is essential to attend to all correctable confounders in clinical trials. In this regard, properly designed RCTs could minimize confounding effects, such as subject variability, through randomization (using other mechanisms like stratified randomization), which distributes potential confounders evenly across treatment groups. While a proper randomization process can significantly reduce study confounders for pharmaceuticals, it does not apply to micronutrients. Faulty study designs can overwhelm the validity of data and conclusions by amplifying confounders.

Minimizing confounders in observational studies can be achieved by increasing the sample size and the duration of the clinical study [43]. In contrast, the varied circulating 25(OH)D concentrations observed in both RCTs and observational/ecological studies may also reflect behavioral factors (e.g., taking supplements or consuming other medications) or non-vitamin D-related mechanisms, such as variable solar UVB exposure and ambient UVB dosage, as reported in cardiovascular diseases (CVDs) [42,48,50].

In addition to the above, some confounding factors could modify the relationship between vitamin D and cancer prevention and mortality [43]. For instance, individuals with lower vitamin D status may exhibit generally unhealthy (or risky) behavior, sedentarism, and sub-optimal nutrition, such as reduced physical activity and obesity, which could independently influence cancer risk. Limited outdoor activity and lower sun exposure reduce vitamin D synthesis [47]. These interrelated factors complicate the attribution of cancer prevention effects to micronutrients, specifically vitamin D [43].

Correlating micronutrients like vitamin D with multiple clinical benefits beyond diseases such as rickets and osteomalacia is challenging but feasible. Such difficulties are partly due to technical and methodological issues [54]. For vitamin D, it is impossible to have a true placebo group in RCTs due to the widespread consumption of over-the-counter supplements and variable exposure to ambient UVB rays [43,53]. Unlike pharmaceutical agents, vitamin D has a threshold beyond which a demonstrable beneficial effect is not observed [55,56], except in cases of vitamin D-resistant syndromes [49].

### 1.3. Vitamin D—Cancer Risk Reduction vs. Mortality

When designing clinical trials related to micronutrients, several factors must be considered to minimize confounders, thereby allowing for improved clinical data interpretation, as recently emphasized in *Nutrient Reviews* [43]. The most robust associations between vitamin D and cancer outcomes pertain to mortality rather than incidence [57,58,59,60]. This distinction arises partly because, unlike symptoms (e.g., morbidities), mortality is a definitive (accurate) and consistently measurable endpoint.

In contrast, cancer incidence can be influenced by various controllable and independent factors, including screening practices, sensitivity of the methods used, diagnostic criteria, reporting standards, healthcare access, and social determinants, which lead to variability in detection timelines [50,53]. Consequently, it is unsurprising that the mortality data provides a more reliable basis for assessing the potential benefits of vitamin D supplementation in cancer-related outcomes, as well as in other conditions like cardiovascular and pulmonary disorders. However, that does not exclude the beneficial effects of vitamin D in disease prevention.

Additionally, studying the role of vitamin D in cancer prevention and/or mortality is even more challenging, given the need to select appropriate subjects and conduct multi-year follow-ups with proper vitamin D supplementation. However, such studies suffer from poor compliance and a high incidence of loss to follow-up. These factors significantly affect data collection, interpretation, and clinical outcomes, thereby impacting the validity and generalizability of the findings to other populations.

***Mendelian analyses:*** While valuable in specific contexts, Mendelian randomization studies and analyses often fall short of establishing causation and hold limited significance for nutrients like vitamin D [61]. Such analyses consequently fail to provide helpful information about vitamin D and are therefore not recommended, as they are too far from the incident [50]. Thus, they infrequently provide conclusive evidence of a causal relationship between circulating 25(OH)D concentrations and cancer-related health risks [53,62]. Moreover, it is important to note that meta-analyses—often susceptible to selection bias—have also produced negative or inconsistent results, particularly when they include flawed RCTs, thereby affecting the validity of their conclusions [63].

### 1.4. Systematic Review Process

This systematic review (SR) was conducted following the Preferred Reporting Items for Systematic Reviews and Meta-Analyses (PRISMA) guidelines over a defined period [64,65,66] and the Participants, Intervention, Comparison, Outcome (PICOS) framework (Table 1) [67]. The author followed guidance from the Equator Network (www.equator-network.org/, accessed on 15 November 2024) and the PRISMA statement, as well as the PRISMA-P checklist [61,64,68], to assess the quality of the literature [67]. PRISMA Checklist [66] is provided in Supplementary Materials.

#### 1.4.1. Literature Search

Researchers (SJW and HB) systematically searched PubMed, Medline, EMBASE, and the Cochrane Central Register of Controlled Trials for original and review articles on vitamin D status in the general population, including broader aspects. The search, using terms such as “vitamin D,” cholecalciferol, 25(OH)D, and 25-hydroxycholecalciferol, in conjunction with cancer risks and mortality, resulted in 3995 peer-reviewed publications related to vitamin D and cancer or malignancy.

#### 1.4.2. Rationale for the Study

Although the effects of vitamin D on skeletal tissues are well established, its biological functions in non-skeletal tissues are less understood. Over the past two decades, thousands of studies conducted in extra-skeletal systems have reported mostly positive but some reported non-conclusive effects of vitamin D. Despite these, vitamin D has overwhelmingly significant biological and physiological effects on extra-skeletal tissues. For example, related to cancer, calcitriol plays a crucial role in regulating cell proliferation, differentiation, and apoptosis, as well as controlling the release of cytokines, growth factors, hormones, and cellular signaling [69,70,71,72]. These effects are vital for cancer prevention, metastatic progression, and death. As a result, vitamin D is considered an essential factor in regulating cell growth and differentiation, thus suppressing the risks and development and progression of cancer.

However, the precise mechanism by which calcitriol exerts its physiological effects on extra-skeletal tissues could be deeper than we currently understand. Numerous recent studies have examined the relationship between vitamin D and cancer, mostly on mortality. This SR focuses on assessing the effectiveness of vitamin D in cancer prevention [73] and its role in reducing the spread and mortality. 

#### 1.4.3. Objective of the Study

The current understanding of vitamin D biology and physiology is primarily based on retrospective analyses, case reports, and epidemiological studies [27,74,75]. Although numerous studies have explored the relationship between vitamin D and cancer, only a few RCTs have focused on cancer prevention [76], including the effects of ultraviolet B (UVB) rays [77]. However, most of them have used insufficient doses to raise serum 25(OH)D to therapeutic levels of above 40 ng/mL Despite the lack of complete consensus, most evidence supports the paradigm that adequate vitamin D reduces the risk of certain cancers (the biology of each cancer can be different) and mitigates their severity and metastasis [78,79,80,81].

Most studies support maintaining serum 25(OH)D levels higher than 40 ng/mL to achieve vitamin D’s metabolic benefits, including reduced cancer risk [40,41,82] and mortality [83,84]. Even greater protection is reported at levels above 50 ng/mL [41]. However, some recent RCTs, especially those that relied on serum 25(OH)D levels less than 30 ng/mL, have yielded inconclusive or conflicting results [42,45,46,85,86]. In addition, such reports are primarily due to poor study designs, including the inclusion of non-deficient participants, failure to measure baseline levels, and failure to achieve the predefined therapeutic 25(OH)D levels after supplementation [42,85,86].

Future clinical research should prioritize well-designed ecological studies. If RCTs are to be used, they must be unbiased, adequately powered, and based on rigorous, appropriate study designs [3], focusing on physiological mechanisms to clarify how vitamin D reduces cancer risks and mortality [70,87,88,89,90,91,92,93,94]. In this SR, key data from relevant scientific publications on vitamin D and cancer were collected, synthesized, analyzed, and interpreted. The primary aim was to evaluate how vitamin D influences cell growth in reducing cancer incidence, severity, metastasis, and mortality, and to determine whether it lowers overall cancer risk and death rates. 

#### 1.4.4. Search Strategy

PubMed, Medline, EMBASE, and the Cochrane Central Register of Controlled Trials were searched systematically for prospective original studies, epidemiological data, and reviews for this systematic review. Included articles focused on vitamin D as the primary supplement, its mechanisms of action on cancer, and related clinical outcomes. Controlled search terms included “vitamin D,” cholecalciferol, 25(OH)D, 25-hydroxycholecalciferol, calcifediol, calcitriol, cancer risks/incidence, cancer mortality, and malignancy. Additional terms, such as 25-hydroxyvitamin D (25(OH)D), calcifediol, and calcitriol, were selected from Medical Subject Headings (MeSH) and the EMTREE thesaurus [8], combined with “cancer” and ‘malignancy” to refine and manage search volume.

#### 1.4.5. Protocol and Manuscript Selection

A protocol was developed to streamline and track relevant publications. The SR includes observational studies, epidemiological investigations, randomized trials, and mechanistic or hypothesis-generating studies [65], all of which are relevant to the topic. The literature search involved regular original papers, updates, and full-text reviews [64], with article selection based on predefined criteria for cancer and vitamin D.

**Figure 1:**
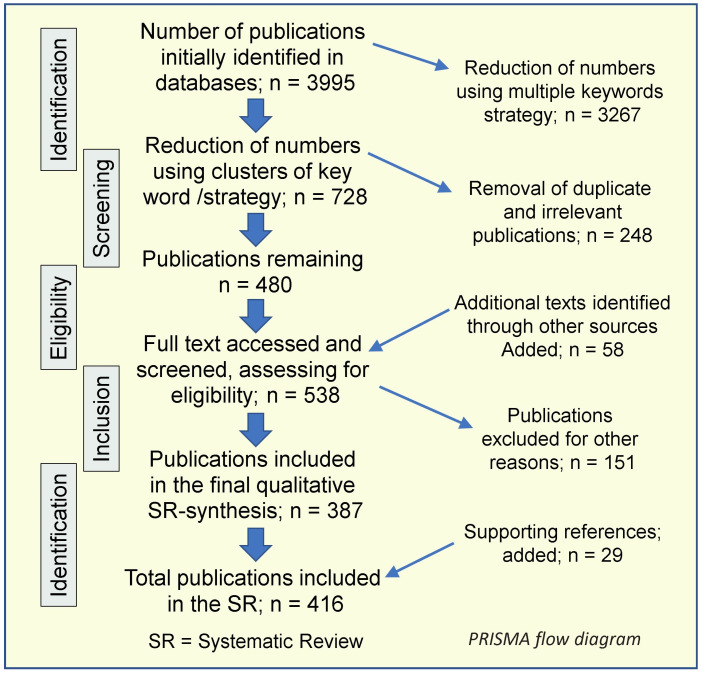
PRISMA flow chart—Flow chart of the assessment of research papers and advances in the knowledge of vitamin D [levels of 25(OH)D] in, modifying cancer risks, metastasis, and death rates (SR = systematic review).

This review rigorously evaluated selected studies using conceptual frameworks aligned with their objectives. It considers peer-reviewed, English-language articles published between January 1991 and March 2025. The initial database screening identified 480 articles after removing duplicates and irrelevant entries. Secondary and tertiary further EndNote searches (version 21.4, Thomson Reuters)retrieved 87 additional full-text articles. This SR included 416 articles. Figure 1 outlines the PRISMA review process [66].

#### 1.4.6. Data Abstraction and Synthesis

Assessments considered each study’s rationale, objectives, design, potential biases, and eligibility criteria. Data collection, analysis, and synthesis followed the evidence-based PICOS framework (Table 1) [67] and included meta-analytical approaches, as undertaken previously when applicable [66]. Irrelevant and duplicate articles were excluded. Researchers critically appraised the strength of evidence on vitamin D’s role in cell proliferation, cancer development, and metastasis.

Furthermore, data were reviewed and independently evaluated according to assigned tasks by two investigators. Reviewers performed specific analyses and resolved disagreements through discussion. Integrated data were presented as narrative conclusions [64] that integrate evaluations from observational and ecological studies, as well as RCTs, and apply the National Heart, Lung, and Blood Institute quality assessment tool.

#### 1.4.7. Literature Search and Analytical Outcomes

This SR assessed and highlighted the global prevalence of vitamin D deficiency, particularly among individuals with chronic illnesses [34], including cancer [95,96,97], and in children [98], in alignment with its theme. Cancer incidence continues to rise, particularly in regions with limited sunlight [31,98,99] and in areas characterized by sun-avoidant behaviors [31,95]. Approximately one-third of the publications reported inconsistent or no significant effects of vitamin D supplementation on cancer, often due to inadequate dosing or short intervention periods [42,45,46,85,86]. Only one-third of the remaining studies focused on the role of vitamin D in cancer prevention.

Furthermore, this SR emphasizes the urgent need for higher-quality, well-designed, longer-term prospective and ecological clinical studies, as well as RCTs (although less suitable to test efficiencies of micronutrients like vitamin D) that are adequate and statistically powered and which are provided with sufficient vitamin D (i.e., a minimum of 5000 ID/day, preferably following a loading dose of about 200,000 IU stat dose) and duration to test hypotheses regarding the effects of vitamin D on cancer. Such studies are very few, and thus, more are needed. Many recent large-scale sponsored RCTs have been flawed by preventable design errors [42,45,46,85,86].

Mentioned design errors include enrolling participants with adequate baseline vitamin D levels, failing to assess dose–response relationships or account for daily sunlight exposure, not measuring baseline 25(OH)D concentrations, and failing to assess the baseline serum 25(OH)D levels and correlate the achieved serum 25(OH)D levels (instead of doses administered) with clinical outcomes [43,53] [48]. Additional issues, such as permitting participants to use over-the-counter micronutrients (including vitamin D), not defining target serum 25(OH)D levels, and omitting hard clinical endpoints, misperceive results and conclusions [1].

Studies that have reported negative or inconclusive outcomes commonly exhibited one or more of these fundamental methodological shortcomings. In contrast, nearly all well-designed, adequately controlled, and statistically powered RCTs have consistently demonstrated that adequate vitamin D supplementation significantly reduces cancer risk and mortality [96,97,100,101].

#### 1.4.8. Scope of This Review and Outcomes

In addition to the global rise in vitamin D deficiency and the cancer incidence discussed in Section 1, in many countries, there is a lack of awareness and implementation of practical cancer prevention guidelines [27,102]. Furthermore, recommendations from scientific societies are often contradictory, and guidance on sun exposure remains inconsistent [103,104]. It is crucial to ensure the long-term maintenance of serum 25(OH)D concentrations above 40 ng/mL [49]—the minimum effective level—with consideration for age, body weight, or for BMI-tailored optimal vitamin D doses [40,49,55,56,105].

The reported large effect sizes in clinical studies that have used vitamin D in cancer suggest hypovitaminosis as an essential factor contributing to vulnerability, particularly in cancers like breast and colon [106,107,108,109,110,111]. Given these considerations, along with the high cost of care, morbidity, and mortality, as well as ethnic differences in cancer incidence and outcomes, individual countries or regions (e.g., Africa, the Gulf, North America, and Southeast Asia) must develop targeted guidelines for their respective populations. When properly applied, these guidelines could significantly reduce the risks of cancer, chronic diseases, cardiovascular disorders, viral infections, autoimmune conditions, and other health issues.

The above considerations should include the costs and benefits of raising awareness across populations to ensure the use of recommended dietary allowances (RDAs) for micronutrients, including vitamin D. Establishing safe and effective serum 25(OH)D ranges (minimum and upper safety limits), safe sun exposure guidelines and directives for targeted food fortification programs are cost-effective and particularly valuable for vulnerable populations. A proactive approach to maintaining long-term vitamin D sufficiency—maintaining serum 25(OH)D concentrations above 40 ng/mL—will reduce the burden of chronic diseases, including cardiovascular, metabolic diseases, and cancer risks [95,96,97]. While this SR focused on vitamin D’s role in cancer prevention, the same procedure and principles apply to other chronic diseases [34] and help mitigate viral respiratory epidemics and pandemics [112,113].

## 2. Vitamin D Requirements—Sun Exposure, Biological Functions, and Cancer

A recent narrative review found that solar radiation reduced the risks and mortality of 23 types of cancer and showed stronger inverse correlations between serum 25(OH)D levels and 12 cancer types. These findings were based on observational studies, meta-analyses, and case-control studies. Unlike Mendelian randomization studies, serum 25(OH)D concentrations were measured in these studies, closer to the time of cancer diagnosis [87]. Clinical studies have also found that daily or weekly vitamin D intake has a greater effect on reducing cancer risk than less frequent dosing, such as monthly [49,114,115]. Despite extensive data, there is no consensus on optimal serum 25(OH)D levels for reducing cancer risks. A detailed formula and tables for calculating daily vitamin D intake based on body weight or BMI for individuals have been published [82]; a simplified version is provided below [33].

I. Not obese (average wt.: BMI, <29): 70–90 IU/kg BW
II. Moderately obese (BMI, 30–39): 100–130 IU/kg BW
III. Morbid obesity (BMI, over 40): 140–180 IU/kg BW

Current vitamin D standards and government guidelines are primarily based on outdated RCTs and focus solely on the minimum requirement for skeletal health—namely, prevention of rickets. They have mistakenly ignored the need for all other body systems, and focus solely on the minimum requirement for skeletal health [116]—namely, prevention of rickets [27,74,75], a limitation that has been noted by others [117,118]. In contrast, over the past fifteen years, robust evidence has emerged supporting the benefits of vitamin D outside the skeleton, including its role in cancer prevention [81,119,120,121,122]. Additional studies have shown the role of vitamin D in regulating cell growth and differentiation [123], inhibiting cancer progression, and reducing mortality [83,84].

In the absence of adequate sunlight exposure, maintaining a blood level of 25(OH)D that is above 40 ng/mL (75 nmol/L) in “non-obese” individuals will typically require a daily oral intake of at least 5000 IU (125 µg) of vitamin D_3_. For these individuals, the safe upper limit for long-term vitamin D use is recognized as 10,000 IU per day [124,125,126,127,128]. Meanwhile, the recommended minimum serum 25(OH)D concentrations ranged from 30 to 60 ng/mL [41], while higher levels are more effective. For example, evidence strongly suggests that levels above 50 ng/mL are necessary and better to effectively combat cancer, infections, heart disease, and autoimmunity, and to support robust immune functions [55,56,129,130,131]. The overall research data from positive trials suggest that the minimum level necessary for cancer risk reduction and reduced mortality is 40 ng/mL [40,105]. 

### 2.1. Sun Exposure and Generation of Vitamin D

Despite the health benefits of safe sun exposure, generating sufficient vitamin D_3_ among populations has limitations. Sun avoidance can markedly reduce the dermal production of vitamin D. The earth’s atmosphere absorbs (and reflects) UVB radiation, leading to a lesser amount reaching the surface [132]. Factors include sun avoidance behavior, darker skin pigmentation, time of day, and duration of sunlight exposure [133,134]. When evaluating sun exposure, the month and season are important. The solar zenith angle—the angle between the sun and the vertical axis—particularly during early mornings, evenings, and winter, results in less UVB penetrating the skin [77].

Ecological studies have demonstrated a significant inverse correlation between solar UVB exposure and the incidence of certain cancers and cancer-related mortality [70,87,88,89,90]. One population-based study suggested that achieving a serum 25(OH)D concentration sufficient to reduce cancer risks through casual sun exposure would require an oral intake of approximately 2800 IU of vitamin D per day [135], perhaps an underestimation. This highlights the pressing need for public health strategies that prioritize vitamin D sufficiency. By addressing the challenges posed by sun exposure limitations and evolving dietary guidelines, healthcare systems can mitigate the prevalence of hypovitaminosis D cost-effectively. Simultaneously, research into optimal supplementation protocols and their integration into preventive healthcare practices will serve as a crucial step toward reducing the global burden of chronic diseases and cancer [136,137].

### 2.2. Causal Role of Vitamin D Deficiency in the Development of Select Cancers

Studies applying Bradford Hill’s criteria for causality have confirmed that hypovitaminosis D significantly increases vulnerability to various diseases [49,53,109,119,138]. Several other studies have provided compelling evidence that hypovitaminosis D is a major factor contributing to developing complications and increased mortality from COVID-19 [55,56,138,139], as well as increasing the risk of other diseases such as multiple sclerosis and periodontal disease [55,56,138,139], including cancers [87]. However, in certain areas, evidence from RCTs remains weak or inconclusive, with a few studies reporting negative outcomes [75,140,141]. Poor clinical study designs limited the proper interpretation and value of these studies [51,52].

Findings from the current study should be used to expand knowledge among healthcare workers and scientists, aiding in the distribution of essential information regarding the relationship between serum 25(OH)D concentrations and cancer risks. The data support that hypovitaminosis D significantly increases the vulnerability to cancer [49,142]. Conversely, maintaining serum 25(OH)D concentrations above 40 ng/mL [27,102] appears promising in controlling cancer growth and metastasis, as well as reducing motility [40,41,143]. Studies in Western Europe have shown that population-based vitamin D supplementation strategies may reduce the economic burden by decreasing the prevalence of cancer and other chronic diseases [20,34,35,144]. 

### 2.3. Vitamin D Plus Calcium—Effect on Cancer

While two RCTs have reported that vitamin D plus calcium supplementation had no benefit in reducing cancer risk [89], reanalysis of data from cohorts such as the GrassrootsHealth volunteer group—based on achieved serum 25(OH)D concentrations rather than administered dose—demonstrated a significant reduction in breast cancer risk [41]. Women with 25(OH)D concentrations ≥60 ng/mL had a substantially lower risk compared with those with levels <20 ng/mL (95% CI, 0.04–0.62; *p* = 0.006) [41]. More information is provided in a Q&A [102,145]. 

A similar inverse association with breast cancer incidence has been observed across three independent cohorts, indicating that higher serum 25(OH)D concentrations are linked to a lower risk of breast cancer. [146]. However, other studies have reported inconsistent findings [114]. Moreover, the data indicate that, while vitamin D alone may benefit, adding calcium provides limited advantages and may even lead to adverse effects.

The seven-year Women’s Health Initiative (WHI) trial found that daily supplementation with 1000 mg of calcium and 400 IU of vitamin D_3_ did not significantly reduce the overall incidence of invasive cancers in postmenopausal women [147,148]. However, despite the low dose and the permitting of participants to take additional over-the-counter supplements, a post-hoc analysis revealed a significant reduction in breast and colorectal cancer incidence among women who were using vitamin D and calcium supplements at the time of recruitment [149,150]. 

In the WHI study, women who received calcium and vitamin D supplementation, particularly those who were vitamin D deficient before the trial, experienced a significantly lower incidence of breast cancers (a 14% to 20% risk reduction) and a nonsignificant 17% reduction in colorectal cancers [109,151]. Cancer risk reductions were seen mainly in post-menopausal women who had low vitamin D levels at the start. Other studies have reported no significant risk reduction for all cancer types with combined calcium and vitamin D supplementation [93,152].

However, reported findings related to vitamin D, calcium supplementation, or their combination, and the dose–response relationships with health outcomes were inconsistent [153]. In many of these RCTs and reviews, vitamin D alone showed protective effects against cancer, but these benefits diminished or disappeared when combined with calcium [154]. Poorly designed studies—such as several large cancer trials using vitamin D, including the VITAL [155] and D2d studies—unsurprisingly reported negative outcomes, as discussed in Section 4.1.

An exception is dietary sources like milk products, particularly cheese [156], which contain both vitamin D and calcium and are associated with a reduced risk of colorectal cancer [157,158]. In contrast, well-designed clinical studies that supplemented subjects with adequate doses of vitamin D to influence endpoints consistently reported significant benefits, including reductions in cancer risk and mortality.

### 2.4. Vitamin D 1,25(OH)_2_D Interactions and Cell Proliferation

Interactions between 1,25(OH)_2_D] and vitamin D receptor (VDR) occur in virtually every cell in the body [27,102], including cancer cells. Vitamin D influences the transcription of cell cycle proteins, resulting in a reduced rate of cell proliferation [159,160,161]. Moreover, an elevated vitamin D status enhances cell differentiation in various cell types, including osteoclast precursors, enterocytes, keratinocytes, gastrointestinal epithelial cells, and precancerous and cancer cells [160,162].

Evidence from RCTs and ecological studies suggests that maintaining serum 25(OH)D concentrations above 30 ng/mL (but significantly improved outcomes when serum 25(OH)D levels are maintained higher than 40 ng/mL) is associated with a reduced risk of some cancers. However, this may not impact cancer severity, spread, or mortality [89,144,163], perhaps because serum 25(OH)D concentrations are below the threshold needed. Another study reported a significant decrease in cancer incidence when the mean serum 25(OH)D concentration increased from 33 ng/mL to over 45 ng/mL [39]. Most studies have demonstrated an inverse correlation between serum 25(OH)D concentrations and cancer risk [87,164,165,166]. However, it remains uncertain whether normalizing vitamin D status can reduce the risk of progression or dissemination of existing cancers or whether this applies to all cancer types [39,167,168].

Meanwhile, clinical studies on colorectal cancer have confirmed a strong inverse correlation between dietary vitamin D and calcium intake and cancer risk [150,169,170]. However, other studies have reported conflicting findings [147], primarily due to methodological differences. Unsurprisingly, a meta-analysis of RCTs has indicated that vitamin D supplementation at doses between 400 and 1100 IU/day, even when administered for up to seven years, had a minimal impact on cancer incidence but was associated with a reduction in total cancer mortality [171]. This further underscores the importance of using higher doses and maintaining elevated serum 25(OH)D concentrations than currently recommended. 

### 2.5. Effects of Vitamin D on Cell Proliferation and Metastasis

Vitamin D lowers cancer risks, among others, by inhibiting cell proliferation, promoting differentiation and apoptosis, and suppressing angiogenesis [106]. Vitamin D suppresses angiogenesis by downregulating pro-angiogenic factors, such as hypoxia-inducible factor-1 [172,173] and vascular endothelial growth factor (VEGF) [174,175]. Vitamin D also regulates angiogenesis through NF-κB signaling, which induces angiogenic factors, such as IL-8 and VEGF [175], as reported in prostate cancer cells [176] and suppression of prostaglandin pathways [177]. Additionally, in melanoma cell lines, vitamin D_3_ mediates an anti-proliferative effect and modulates the expression of key cell cycle regulatory molecules, such as p21, p27, cyclin D1, and cyclin A1 [178].

Inhibiting metastasis involves reducing proteases, such as matrix metalloproteinase 9 (MMP9), matrix metalloproteinase 13 (MMP13), and cathepsin, that degrade the extracellular matrix. Vitamin D also upregulates protease inhibitors, such as tissue inhibitors of metalloproteinase 1 (TIMP-1) and cathepsin inhibitors, which prevent the degradation of the extracellular matrix [179,180]. In addition, suppressing angiogenesis, CDK2, and stimulating p21 reduces the metastasis of cancer cells [181].

The mechanisms by which vitamin D reduces cancer incidence include its regulatory effects on cellular differentiation, proliferation, and apoptosis (programmed cell death). Additionally, the observed reductions in cancer metastasis and mortality are linked to vitamin D’s ability to inhibit angiogenesis (formation of new blood vessels) within tumors [106,175] and surrounding tissues [87], as well as its modulation of MMP9 and MMP13 and cathepsin, enzymes involved in extracellular matrix degradation [180,181].

### 2.6. Vitamin D Sufficiency–Protective Against Cancer

The incidence and severity of breast cancer are inversely associated with serum 25(OH)D levels, especially in post-menopausal women [106,107]. In addition, meta-analyses have reported the improved survival of persons with breast cancer when they maintained higher circulatory 25(OH)D concentrations [108]. Using similar data sets, however, others reported no benefit from vitamin D in breast cancer risk reduction [45,93,94,149,152,182,183,184]. 

Hypovitaminosis D has been associated with an increased risk of developing and dying of certain cancers [185,186,187]. However, maintaining serum 25(OH)D concentrations above 40 ng/mL significantly reduces the risks of cancer invasion [40] and cancer-related mortality [40,188]. Individuals residing at higher latitudes face an increased risk of developing and dying from common cancers [189,190], including those affecting the colon, breast, and prostate [189,190,191,192,193,194,195,196]. Conversely, increased exposure to solar UVB radiation is associated with decreased risks of developing and succumbing to breast, colon, ovarian, and non-Hodgkin lymphoma cancers [197,198].

### 2.7. Effectiveness of Vitamin D in Different Cancer Types

Numerous theories have been suggested to explain how vitamin D reduces cancer risks [166,198,199,200]. These include the impact of adequate intracellular calcitriol levels, which increase the expression of protective genes. Additionally, calcitriol inhibits tumor progression and enhances survival, particularly in tumors exhibiting high vitamin D receptor (VDR) expression [201,202]. Calcitriol increases VDR expression in immune cells [203,204]. Table 2 outlines diverse aspects of cancer along with corresponding references.

### 2.8. Ultraviolet B, Vitamin D, and Prevalence of Cancer

Research indicates that exposure to ultraviolet B (UVB) rays reduces the risk of various types of cancer [1,163,289,290,291,292]. Calcitriol has been shown to enhance the expression of protective microRNA-22 in colonic cells, thereby reducing colon cancer cell proliferation [293]. Vitamin D sufficiency also supports healthy intestinal microbiota [294], which may help reduce the risk of colon cancer [295]. Additionally, an inverse correlation has been noted between UVB radiation and mortality in individuals with several malignancies, including bladder, esophageal, kidney, lung, pancreatic, rectal, stomach, and corpus uteri cancers [195], often linked to inadequate sun exposure, particularly at higher latitudes [261,262,263].

Research from Nordic countries, including Sweden [296], has also shown an inverse association between UVB exposure and cancer risk [163,289,290]. Higher UVB exposure is also correlated with longer life expectancy [297]. However, it remains unclear whether this is solely due to UVB or influenced by other healthy lifestyle factors [292]. For example, people in higher latitudes of Europe consume more fish and meat, which contain 25(OH)D, acting as a confounding factor [298,299]. They also tend to lead healthier lives and consume more fatty fish, thereby increasing their omega-3 fatty acids and vitamin D [300], which may reduce cancer risk and mortality, as well as enhance longevity [166,297,299,300].

### 2.9. Sun Exposure, Genetics, and Skin Cancer

Ultraviolet radiation, a component of the electromagnetic spectrum naturally emitted by the sun or generated artificially (e.g., through tanning devices), can induce exposure-dependent skin reactions. These reactions include erythema, sunburn, skin wrinkling, and DNA damage to dermal cells [284,301]. Prolonged and frequent exposure to ultraviolet radiation is a primary factor contributing to skin cancers, including cutaneous malignant melanoma, basal cell carcinoma, and squamous cell carcinoma [103]. Consequently, avoiding excessive exposure to sunlight may lead to sunburn [1].

Skin cancers are common among individuals with white skin who have freckles. This skin type is more vulnerable to developing, representing this group’s most frequent genetics-related skin cancer type [301]. In addition, excessive sun exposure during childhood can cause lasting damage, with adverse effects appearing later in life. The risk is higher in those with freckled skin [77], suggesting underlying genetic susceptibility. The majority of sun-induced skin cancers are observed in individuals residing in areas where their skin phenotype is not optimally adapted to elevated levels of ultraviolet radiation, for example, people of European descent living in Australia. 

Over generations, these fair-skinned individuals, whose ancestors migrated from central Africa to regions such as Europe, the United States, and later to Australia and New Zealand, have adapted to having lighter skin to generate more vitamin D from lesser UVB exposure. However, when exposed to higher duration and intensity of UV rays, they face a heightened risk of developing skin cancer [77]. People with darker skin in Africa do not use sunscreen, yet have lower rates of skin cancer and melanoma [301].

### 2.10. Sun Exposure Reduces Cancer Risks

Several epidemiological studies have linked reduced sun exposure—and the resulting low serum 25(OH)D concentrations—to higher incidences of breast cancer [224,225,226]. Researchers have observed similar correlations in colon, prostate, and ovarian cancers, as well as in non-Hodgkin lymphoma and certain types of leukemia [302,303]. Additional studies have reported strong inverse associations between UVB exposure and the risk of ovarian [304] and esophageal cancers [87,305]. These findings align with the observed increase in cancer prevalence among people living in northern latitudes, where sun exposure is limited [89,265,266,267].

Additional data suggest that individuals regularly exposed to sunlight are less likely to succumb to cancer [31,99,119]. Consequently, the findings indicate that the serum is maintained at a higher level. The best strategy to ensure adequate 25(OH)D levels is to promote reasonable, intentional sun exposure, recognizing individual variability in requirements. Applying sunscreen after sun exposure helps prevent sunburn while allowing benefits [284,306]. Meanwhile, individuals should avoid tanning beds, as they can lead to excessive exposure to both UVB and UVA radiation. The latter could increase cancer risk and also accelerate vitamin D catabolism [307]. It remains unclear whether these benefits arise solely from raising and maintaining circulating 25(OH)D levels or if sun exposure provides additional protective effects against cancer.

Recurrent inflammation following sunburn has the potential to trigger a detrimental cycle, culminating in skin fibrosis and an increased risk of cancer [301]. This risk is particularly notable in individuals with lighter skin tones who are genetically predisposed (e.g., those with freckles, as noted above). Notably, consistent exposure to sunlight, including among those who work outdoors or participate regularly in outdoor physical activities [308,309], has been reported to diminish the risk of melanoma [1,252]. This observation suggests that the risk of melanoma does not necessarily increase with sun exposure [281,282,283].

### 2.11. Additional Mechanisms of Vitamin D in Cancer Risk Reduction

Calcitriol has been shown to reduce cell proliferation and induce apoptosis [310], promote autophagy, and inhibit the growth of cancer cells. It also modulates the immune system to counter cancer progression, indicating that vitamin D suppresses cancer growth and metastasis [272]. Additional mechanisms involve 1,25(OH)_2_D-mediated reductions in proliferation, angiogenesis, and growth, alongside enhanced differentiation, and anti-inflammatory effects [84,209]. These pathways collectively increase apoptosis and reduce cancer cell metastasis [285,287,288].

Some of vitamin D’s beneficial effects are mediated through the stabilization of mitochondrial functions and suppression of oxidative stress (reactive oxygen species) via multiple mechanisms [311]. Furthermore, calcitriol plays a crucial role in intracellular calcium mobilization, which has been linked to the pathophysiology of various extraskeletal conditions, including cancer cell growth and metastasis [156,191]. Vitamin D regulates oxidative stress, inflammation [312,313,314], and energy metabolism [75,315]. Inflammaging and oxidative stress are linked to cancer [316]. These mechanisms and enhanced DNA repair [317] are crucial for reducing the risk of cancer.

Additionally, vitamin D metabolism is influenced by medications, environmental pollutants that affect the cytochrome P450 system, and lifestyle factors such as physical activity (sedentary vs. being active) [300,308,309], which impact energy balance [318]. When designing clinical studies, including RCTs, and managing patients, healthcare professionals must consider these factors and tailor their approaches to optimize patient outcomes. In addition, specific VDR gene variants or polymorphisms may influence cancer incidence, severity, and mortality [319]. However, as discussed in Section 1.1 and Section 3.4 [256,257,258,259] RCT data are limited and insufficient for firm conclusions. Further studies are needed to understand how polymorphisms in the VDR and other vitamin D-related genes, as well as epigenetic changes, affect cancer risk [256,260,320].

In several diseases, RCT evidence more strongly supports the use of vitamin D for prevention than for treatment [39,151,221,321], whereas in cancer, it is the opposite [91,92,93,94]. Complicating the matter, an RCT in colorectal adenoma reported that the beneficial effect of vitamin D_3_ supplementation varies with VDR genotypes. The risk was found to be reduced in individuals with advanced adenoma who had the VDR rs7968585 AA genotype, while an increased risk was observed in those with the VDR rs7968585 GG/GA genotypes [322]. However, others have reported an inverse association between circulating 25(OH)D levels and colorectal adenoma risk [150], but not with VDR gene polymorphisms (Folk and Bsml) [319].

## 3. Cancer Mortality Relationships

Numerous studies have confirmed a strong association between vitamin D status and mortality from most cancers [91,92,93,94]: cancer risk reductions were less robust. Most observational studies and meta-analyses have reported associations between lower serum 25(OH)D levels and higher overall cancer mortality [91,92,93,94]. A meta-analysis of twelve cohort studies reported a 14% higher cancer mortality in people with the lowest quarter of 25(OH)D levels vs. the highest quarter [92]. Additionally, the Prostate, Lung, Colorectal, and Ovarian Cancer Screening Trial found a 17% lower cancer mortality rate among men and women in the highest quartile of vitamin D intake compared with those in the lowest quartile [323].

### 3.1. Major Challenges Associated with Nutrient Clinical Trials

As described in this section, nutrient clinical trials face several major challenges that limit their reliability and applicability, especially when using methods like RCTs. Unlike pharmaceutical trials, nutrients often exhibit nonlinear dose–response relationships with a threshold effect, delayed or cumulative effects, and strong baseline dependency, meaning benefits primarily occur in individuals with prior deficiencies. Additionally, controlling dietary intake over extended periods is difficult, and ethical constraints prevent researchers from inducing deficiencies to establish controls. RCTs, while they used to be considered the gold standard in drug testing, are often poorly suited for studying nutrients due to their complex interactions with genetics, environment, lifestyle, and co-nutrient status. These factors, combined with funding limitations and variability in nutrient bioavailability, complicate trial design, data interpretation, and generalizability of results.

### 3.2. The Factors Hindering Large Vitamin D RCTs from Generating Meaningful Data

Recently published large RCTs related to vitamin D, such as the VITAL study (2000 IU/day for 5.3 years) [45,324], the Vitamin D to Improve Outcomes by Leveraging Early Treatment (VIOLET) RCT (single dose of 40,000 IU in critically ill patients) [325], the Vitamin D Assessment (ViDA) study (single, monthly doses of 200,000 IU) [326], the D2d study (focused on cancer and pre-diabetes) [327], and the Vitamin D on All-Cause Mortality in Heart Failure (EVITA) study (4000 IU/day) [328], all exhibited significant study design limitations [44,47,49,53]. Like the WHI [151] and VITAL study [45,324], several other large, mega RCTs enrolled vitamin D-sufficient subjects and allowed participants to consume over-the-counter supplements [47], including vitamin D [47,53]. These study designs introduced limitations and misalignments, which significantly reduced the effect size and undermined their statistical power to differentiate between the intervention and placebo groups [43].

Given the above-mentioned significant study design limitations that are present in recent mega-RCTs, their conclusions cannot be relied upon [53]. Furthermore, it is unsurprising that the results of clinical studies have also exhibited variations based on other factors, such as the type of cancer, time of diagnosis, study design, follow-up duration, serum 25(OH)D status, and the inherent biases of investigators [43]. The VITAL study [45] serves as a clear example [155], containing several inherently imperfect design elements that led to misleadingly unfavorable primary outcomes [43]. Studies using sub-optimal vitamin D supplements, such as 1000 IU, to assess the effect of altering the recurrence rate of colorectal adenomas (supplementation of vitamin D_3_, calcium, or both for 3 to 5 years) is another classic error in study strategy that was designed to fail [329]. The design of these studies contains multiple preventable errors, which contribute to and perpetuate erroneous conclusions.

### 3.3. Negative RCTs Do Not Mean That the Nutrient Is Not Efficacious

Many negative RCTs have had design flaws, including improper randomization, insufficient vitamin D doses, lack of serum 25(OH)D targets, allowing over-the-counter supplements, or a failure to focus on vitamin D as the primary intervention (see Section 1.1). As a result, it is not surprising that such studies did not show positive outcomes regarding cancer or other non-musculoskeletal disorders, even with adequate vitamin D [330]. These studies underscore the need for more targeted, hypothesis-driven clinical research to explore the relationship between cancer biology and the effects of vitamin D [43]. In addition, although researchers once considered RCTs part of the hierarchy in clinical trial research methodologies, they do not represent the most appropriate or feasible study design for answering nutritional epidemiologic questions about the long-term effects of specific foods or nutrients [331].

In contrast, epidemiological studies have reported that vitamin D deficiency is associated with a higher risk of certain types of cancer [94,107,108,109,149,169,170]. In addition, numerous epidemiological studies have reported the beneficial effect of vitamin D in reducing cancer risk (incidence) and mortality [94]. A pooled analysis of RCTs and cohort studies suggested that 25(OH)D serum concentrations ≥40 ng/mL are associated with a significant reduction in the risk of various invasive cancers [107]. Therefore, the reported results from studies using vitamin D supplements of less than 5000 IU per day (the failure to increase the dose with body weights higher than 70 kg) and using a minimum serum 25(OH)D level of less than 40 ng/mL should be interpreted cautiously, as they are suboptimal for reducing cancer risk and mortality [43]. Meanwhile, other studies have reported no benefits of vitamin D in cancer [45,93,152,182,183].

### 3.4. Rethinking Research Methods: Limitations of RCTs in Micronutrient Evaluation

Hebert et al. addressed several correctable methodological and design challenges in diet-related clinical research, including limitations in measurement techniques, analytical approaches, and the inherent difficulty of establishing true placebo groups [332]. Additionally, a growing body of robust scientific evidence underscores the fundamental physiological differences between pharmaceutical agents and micronutrients [44,54], emphasizing that RCTs are not the most appropriate methodology for evaluating micronutrient efficacy [331]. Nonetheless, many researchers persist in applying RCT designs to such assessments [43,50]. Several recent reviews have clearly articulated these distinctions [333,334,335,336].

Unlike pharmaceuticals, which typically exhibit linear, dose-dependent responses suited for RCT frameworks [333], micronutrients such as vitamin D follow non-linear, threshold–response curves [49]. Once individuals reach physiological sufficiency, further intake yields little or no additional benefit—an effect that RCTs fail to capture effectively. [43,332]. As Heaney (2014) noted, this threshold behavior, along with other biological and methodological factors, renders traditional RCT and Mendelian randomization designs inappropriate for evaluating micronutrient interventions [333].

RCTs were developed with the primary aim of evaluating pharmaceutical drugs in terms of efficacy and safety. While RCTs remain invaluable in pharmaceutical clinical research, their direct application to nutrient science may not always be appropriate [42,43]. However, over time, the use of RCTs in this context has become more widespread, influenced by established research frameworks and funding models that traditionally emphasize pharmacological interventions [331]. This trend may also reflect a broader reliance on methodologies suited to drug development, which do not always account for the complex, systemic roles of micronutrients in human physiology.

## 4. Broader Outcomes from Vitamin D Clinical Studies

Observational studies are better suited for investigating the biological mechanisms by which vitamin D influences cancer risks (see Section 2.4). Studies indicate that persistent vitamin D deficiency is linked to higher cancer rates [87,136]. Data indicate that maintaining serum 25(OH)D levels above 40 ng/mL through UVB exposure or supplements significantly reduces cancer risks [75,137] and cancer-related mortality [39,41]. Meta-analyses show that increasing vitamin D_3_ intake by 1000 IU per day reduces the risk of colorectal [109] and breast cancer [150,163,229] by 50%. Lower doses, such as 400 IU daily, have also been shown to reduce the risks of pancreatic and esophageal cancers [245] and non-Hodgkin lymphoma [119,246,247]. However, recent studies suggest that most cancers require maintaining serum 25(OH)D levels above 50 ng/mL (preferably over 60 ng/mL) for effective risk reduction [41,83,221].

### 4.1. Effects of Vitamin D on Preventing Specific Cancer Types

Vitamin D has been shown to play a role in reducing the risk of several specific cancer types, including colorectal, breast, and prostate cancers (Table 2). Observational studies and meta-analyses suggest that higher serum 25(OH)D levels are associated with lower incidence and mortality rates for these cancers. Vitamin D may exert its protective effects through multiple coordinated mechanisms, such as the promotion of cellular differentiation, inhibition of proliferation, reduction of inflammation, and enhancement of immune surveillance. RCTs have yielded mixed results—often due to the inclusion of vitamin D-sufficient subjects and suboptimal dosing or vitamin D-sufficient participants. Nevertheless, several post-hoc analyses, even with the VITAL study, have revealed significant reductions in cancer incidence, particularly in non-obese individuals and individuals with low baseline vitamin D status. Outcomes from specific types of cancer are described below.

***Breast cancer:*** Several studies with improved designs have shown a significant inverse relationship between serum 25(OH)D concentrations and survival among female breast cancer patients [87,215,216,217,218]. Data from ambulatory post-menopausal women across seven consecutive National Health and Nutrition Examination Surveys (NHANES) from 2001 to 2014 indicated that serum 25(OH)D concentrations ≥40 ng/mL were associated with a notable reduction in breast cancer risk [218]. Comparable results have been noted in African American and Hispanic women [228]. Additionally, other studies have reported low serum 25(OH)D levels in individuals with breast cancer [219,220].

***Colorectal cancer:*** Lower 25(OH)D levels at the time of diagnosis of colorectal cancers are associated with higher overall mortality from colorectal cancer [232]. Similarly, findings indicated a 41% lower risk of colorectal cancer in African American women with the highest levels of 25(OH)D compared with those in the lowest quartile [234]. In two studies, greater overall vitamin D intake was associated with a lower risk of early-onset colorectal cancer [337] and all colorectal cancer [109,119,150,233]. 

***Gastric cancer:*** A meta-analysis found no correlation between vitamin D intake or serum 25(OH)D concentrations and gastric cancer. However, a significant inverse association was observed between solar UVB radiation exposure and gastric cancer incidence [87,235]. Conversely, many studies have reported that higher serum 25(OH)D levels are linked to a significantly lower incidence of gastric cancer [236]. Another meta-analysis indicated that serum 25(OH)D levels in the gastric cancer group were significantly lower than in the control group, with low levels associated with poorer clinical outcomes [237]. Additional studies support the association between higher vitamin D intake and a reduced risk of gastric cancer [238]. Collectively, these data suggest that hypovitaminosis increases vulnerability to gastric cancer.

***Thyroid cancer:*** In papillary thyroid carcinoma, lower serum 25(OH)D levels are correlated significantly with poor prognostic factors, such as large tumor diameter and lymph node metastasis [338]. Mean serum 25(OH)D levels at cancer diagnosis were found to be significantly lower (22.4 ng/mL) compared with cancer-free controls (30.1 ng/mL), with a higher incidence observed in African American children [339]. 

**Prostate cancer:** Additionally, a community-based, extensive prospective study with competing risk analysis reported an elevated risk of developing prostate cancer in the highest 25(OH)D tertile (15%) (hazard ratio of 1.35 [95% CI = 1.07–1.70]). Conversely, death rates were high in the lowest 25(OH)D tertile (67%) (HR ratio, 0.79 [95% CI, 0.71–0.89]) [340]. The discrepancy is explained by marked fluctuations of intracellular 1,25(OH)_2_D levels within the prostate and pancreas, rather than elevated levels, which are responsible for the increased mortality rates observed in a limited number of individuals.

### 4.2. Miscellaneous Cancers

In hematologic malignancies, low 25(OH)D levels predict poor outcomes in myeloid and lymphoid cancers, as well as several types of lymphomas. These were linked to unsuccessful autologous and allogeneic transplants [251]. Regarding lung cancer, a meta-analysis indicated that vitamin D reduces the incidence of lung cancers and improves long-term survival [289]. Besides, a prognostic study suggested that the survival benefits of vitamin D supplements were observed in individuals receiving therapies for stage IV lung cancer [241]. The author proposed that these benefits may, in part, stem from alleviating inflammation and depression in these patients.

Nevertheless, dietary and supplemental vitamin D may not reduce the risk of obesity-related cancers [300], but this lack of response is unsurprising. In addition to other obesity-related overriding factors, such as low-grade generalized inflammation, the dose of vitamin D required for individuals with obesity is several times higher than for those with normal body weight or BMI [33,55,146]. Individuals with hypovitaminosis D who developed nasopharyngeal carcinoma are shown to have a significantly higher risk, which is worsened in those with a BMI ≥28 [239]. Hypovitaminosis D also increases the risk of developing oral squamous cell carcinoma from potentially malignant oral disorders [341]. The author suggested modulating immune responses with vitamin D and other appropriate micronutrients as an adjunct therapy to increase survival, prevent recurrences in those undergoing surgery, and reduce adverse reactions to chemotherapy.

### 4.3. Epidemiological and Meta-Analysis Data

A 14-year follow-up study examining cancer incidence in male health professionals using a “vitamin D index” (incorporating both oral intake and vitamin D production) revealed a significant relative risk reduction for esophageal, oropharyngeal, colorectal, and pancreatic cancer. Among various responsive cancers, breast and colorectal cancers met the Bradford Hill criteria for causality [109,119]. At the same time, bladder, esophageal, gastric, gallbladder, ovarian, rectal, renal, Hodgkin’s [342], and non-Hodgkin’s lymphoma provided reasonable supportive evidence [343].

Individuals with breast cancer exhibited a higher prevalence of vitamin D deficiency compared with an age-matched control population, indicating an increased risk of breast cancer associated with hypovitaminosis D [344]. In eleven case-control studies spanning seven countries, inverse correlations have been documented between breast cancer incidence and serum 25(OH)D concentrations [88,321,344]. Additionally, using pooled randomized data, other studies have suggested that serum 25(OH)D concentrations exceeding 60 ng/mL provide the most protective effects [41,83].

Having sufficient 25(OH)D concentrations (i.e., above 40 ng/mL) reduces the risk of cancer and lowers cancer-related mortality [59,137,168,345,346]. For instance, adults who had regular sunlight exposure over two decades and maintained serum 25(OH)D concentrations greater than 20 ng/mL experienced a 30% and 50% risk reduction for colorectal [109,150] and breast cancers [268,269,271,345]. The response rate will likely be higher if serum 25(OH)D concentrations exceed 40 ng/mL [39,40,41].

### 4.4. Correlations of Serum 25(OH)D Levels with Cancer Incidence

Many clinical studies have reported that higher serum 25(OH)D concentrations are associated with risk reductions, as seen in colorectal [109], bladder [347], and breast cancers [106,107,108]. In contrast, the risks of some cancers have shown little association with the lungs and other less common cancers [184,266,348]. Whereas a few cancers, like prostate [349] and pancreatic cancer [243,244], have a potentially detrimental effect of having higher serum 25(OH)D levels. See Section 4.6 for this data.

Another meta-analysis (*n* = 18,808; median age 60 years) of 30 RCTs reported that vitamin D supplementation with a median follow-up, ranging between 1 and 6.2 years, had no significant effect on cancer incidence (RR: 1.03; 95% CI: 0.91, 1.15) or cancer-related mortality (RR: 0.85; 95% CI: 0.70, 1.04) [152]. This SR included several RCTs with short follow-up periods, making it challenging to assess vitamin D′s impact on cancer suppression. In general, lower serum 25(OH)D levels show a strong correlation with increased cancer incidence and other chronic diseases [20,34,35].

Prospective and retrospective epidemiologic studies indicate that levels of 25(OH)D (and D_3_) below 20 ng/mL are associated with a 30 to 50% increased risk of incident colon, prostate, and breast cancer, along with higher mortality from these cancers [172,350,351]. Others have reported that postmenopausal women who increased their vitamin D_3_ intake by 1100 IU reduced their relative risk of cancer by 60 to 77% [89]. Similarly, another study reported a progressive decline of 25(OH)D during the development of cirrhosis into fibrosis and liver cancer [352].

Utilizing annual average erythemal UV doses measured by a NASA satellite, cancers of the bladder, esophagus, colon, gallbladder, prostate, vulva, rectum, Hodgkin’s lymphoma [342], and multiple myeloma were found to exhibit the strongest correlations with UVB exposure [353]. Furthermore, this study reinforced the notion that observational study data, supported by meta-analyses, help establish dose–response relationships for serum 25(OH)D concentrations, demonstrating risk reductions for colorectal [109] and breast cancers [354,355]. Additionally, a significant risk reduction was reported in a meta-analysis of 10 case-control studies (8243 cases and 9697 control subjects) conducted in the United States, Europe, and Australia (the high exposure group, with a confidence interval of 0.63–0.91; *p* = 0.01) [356].

In another study, postmenopausal women who received a daily combination of 2000 IU of vitamin D3 and 1500 mg of calcium reported a 60% reduction in cancer incidence over four years [39,147]. Using Cox proportional hazard regression [HR = 0.7 (95% CI: 0.47–1.02)], the effectiveness of calcium plus vitamin D, compared with a placebo, was suggestive but did not reach statistical significance for reducing cancer risk. Besides, recent data from the same group, using an intention-to-treat analysis, failed to support these findings [39], though the interpretation of this study remains controversial. Notably, online supplementary data demonstrated a significant reduction in cancer incidence among those who sustained serum 25(OH)D concentrations between 50 and 80 ng/mL [39,40,41].

### 4.5. Melanoma and Insulin-like Growth Factor

The incidence of melanoma is higher in individuals with elevated serum insulin-like growth factor-1 (IGF1) concentrations [357]. Notably, the serum IGF-1 concentration positively correlates with dietary fat and protein intake [300] in conjunction with physical activity [299,308], placing those who regularly consume meat at a heightened risk for such cancers [358,359]. Conversely, higher serum 25(OH)D concentrations at the time of melanoma diagnosis are associated with thinner tumors and an increased chance of survival [255]. This suggests that individuals with melanoma or those at an elevated risk of melanoma may benefit from maintaining higher serum 25(OH)D concentrations [103,255].

Vitamin D and its metabolites are used topically to regulate cell differentiation and modulate the immune response. One example is calcipotriene (calcipotriol ointment or cream), used to treat skin disorders, notably psoriasis [102,360]. A retrospective study from Barcelona reported that lower vitamin D levels were independently associated with worse survival in melanoma patients [253]. Another Spanish study found that low vitamin D levels are associated with ulcerations in melanoma and serve as an independent prognostic factor for overall survival in this disease [254].

### 4.6. Prostate Cancer Risks and a J or U-Shape Curve

Several publications, including a meta-analysis, report a potential increase in prostate cancer risk associated with maintaining higher serum 25(OH)D concentrations [361,362,363]. Although controversial, some researchers attribute the reported J-shaped curves to fluctuations in intraprostatic 1,25(OH)_2_D concentrations rather than serum 25(OH)D levels [364,365]. Others argue that elevated serum calcitriol enhances intestinal calcium absorption, which raises intracellular calcium levels in prostatic cells—a risk factor for prostate cancer [366]. Additionally, several studies, including an SR, suggest that higher dairy intake may increase prostate cancer risk [367], though this observation does not clarify why only the prostate appears affected.

Additional studies show that both African American and European American men with prostate cancer who have higher calcium-to-magnesium (Ca: Mg) ratios or consume whole milk face an increased risk of developing aggressive prostate cancer [368]. In another study, men consuming more than 600 mg of calcium per day from dairy products (equivalent to ≤0.5 versus ≥2.5 daily servings) exhibited a 32% higher risk of prostate cancer compared with those consuming less than 150 mg/day (95% CI: 1.08, 1.63) [369].

These findings indicate a possible link between dairy products, due to calcium, and increased prostate cancer risk. However, the specific constituents of dairy products that contribute to this association remain unidentified. It is essential to acknowledge that several additional cancer risk factors, including genetic susceptibility, environmental pollution, exposure to radiation, chemicals, viruses, sedentary lifestyle, and obesity [164], have not been fully considered in these studies.

### 4.7. Prevention of Cancer Risk Reduction

Cardiovascular disease and cancer remain the two leading causes of death in America, together accounting for nearly half of all annual fatalities. While genetic and familial factors may contribute to them, chronic inflammation, oxidative stress—regulated by the immune system—and immune dysregulation increase vulnerability and drive both conditions. Poor dietary habits also play a significant role, fueling not only these two diseases but many others as well. Because numerous natural substances, including nutrients and nutraceuticals, exhibit anti-inflammatory and antioxidant properties, healthcare professionals can use holistic and orthomolecular medical approaches as complementary therapies to lower the risk and mortality associated with cancer [221].

Higher vitamin D levels have been consistently associated with a reduced risk of colorectal cancer [109,347], while lesser effects have been observed in bladder cancer [347]. Meanwhile, other studies have reported no association between vitamin D levels and the risk of breast, lung, and other less common cancers [184,266]. Others have speculated on the differing prevalence of vitamin D deficiency among racial or ethnic groups, suggesting it might partly contribute to cancer disparities [370,371]. 2011–2014 NHANES data report the presence of severe vitamin D deficiency, with a serum concentration of 25(OH)D that is less than 12 ng/mL in 18% of non-Hispanic Black people, 2% of non-Hispanic White people, 8% of non-Hispanic Asian people, and 6% among Hispanic people (4). Meanwhile, African Americans are less likely to use vitamin D supplements than White people in the US [372].

The American Institute for Cancer Research estimates that 30% to 50% of the most common cancers could be prevented through lifestyle modifications [299,373,374]. Table 3 provides examples of these recommendations.

In addition to adhering to these lifestyle guidelines [299] and maintaining a balanced diet [300], physical activity [308] and nutritional therapies [376] can be supplementary in reducing chronic conditions, including cancer risks [309]. These include type 2 diabetes and cardiovascular and chronic respiratory diseases [374,377]. Additionally, such measures can enhance the outcomes and quality of life for cancer survivors. Collectively, these non-communicable diseases, including cancer, are responsible for over 70% of global deaths.

### 4.8. Clinical Trials on Cancer Prevention

Even in the context of the poorly designed VITAL study [45,155,324], after excluding data from the first year (given that it takes several months for such a small dose to increase serum 25(OH)D concentration), there was a notable 25% reduction in cancer incidence observed among individuals with a BMI < 25 and among African Americans [378]. This analysis also revealed a significantly lower rate of cancer-related mortality with vitamin D compared with placebo, as indicated by hazard ratios of 0.79 [95% CI, 0.63 to 0.99] and 0.75 [95% CI, 0.59 to 0.96], respectively.

Numerous peer-reviewed studies support the role of vitamin D in cancer prevention, particularly when serum 25(OH)D levels are maintained above 40 ng/mL [312,355,379]. Garland et al. (2007, 2011) demonstrated that serum 25(OH)D concentrations ≥52 ng/mL were associated with a 50% lower risk of colorectal cancer compared with levels <13 ng/mL [313,314]. Similarly, Lappe et al. (2007) found that daily supplementation with 1100 IU of vitamin D3 plus calcium reduced cancer incidence by 60% in post-menopausal women [89]. A meta-analysis by Vaughan-Shaw et al. (2017, 2021) reinforced these findings, showing that higher pre-diagnostic 25(OH)D levels significantly improved cancer survival [380,381]. These studies demonstrate that achieving vitamin D sufficiency—through sun exposure or supplementation—is a cost-effective and evidence-based strategy to reduce cancer risk and mortality.

These studies have provided overwhelming evidence that hypovitaminosis D significantly increases vulnerability, causing complications and deaths from COVID-19 that fulfill Bradford Hill’s criteria for causality [56,119,138,139]. Other diseases that fulfill these criteria for increased risk of other diseases include multiple sclerosis [55,56,138,139], periodontal disease [55,56,138,139], infection and autoimmunity [8], and cancer [87], particularly against several significant cancer types [87].

## 5. Improving Clinical Outcomes

Maintaining sufficient serum vitamin D and 25(OH)D levels is crucial for the intracellular conversion of these precursors into the active form, 1,25(OH)_2_D (calcitriol). They enable it to exert its intended modulatory effects on mitochondrial activity, enzymatic reactions, signal transduction, and hormone synthesis and secretion in target cells [382]. These effects extend to various systems, including the insulin and parathyroid hormone (PTH), the renin–angiotensin–aldosterone system, and the FGF23–Klotho system.

The evidence strongly suggests that different diseases require distinct serum 25(OH)D concentrations to achieve clinical benefits and prevent sequelae associated with hypovitaminosis D [27,74,75]. Consequently, there is no agreed-upon universal optimal serum 25(OH)D concentration that ensures all beneficial outcomes [75,383]. Consequently, there is no agreed-upon universal optimal serum 25(OH)D concentration that ensures all beneficial outcomes. Insights from metabolomics, transcriptomics, and adequate supplementation studies promise better information on longer-term extra-skeletal benefits. Additionally, adequate vitamin D supplementation offers the potential for personalized, targeted interventions to mitigate skeletal and soft tissue health risks cost-effectively [19,384].

Earlier studies on vitamin D and CVD, based on older protocols with lower supplementations, such as 2000 IU/day, have been reported to reduce the risk of CVD and related mortality [385,386]. However, more recent evidence indicates that higher daily doses—particularly above 5000 IU—and maintaining serum 25(OH)D concentrations between 40 and 80 ng/mL yield better clinical outcomes than said lower daily doses [50]. That includes higher doses providing greater protection—lower risks, and mortality rates from CVD and other chronic diseases [33,34]. The same principles apply to vitamin D and cancer [321,387,388,389].

In the absence of adequate exposure to sunlight, raising and maintaining blood levels of 25(OH)D above 40 ng/mL (75 nmol/L) in most individuals will require a daily minimal oral intake of 6000 IU (125 µg) of vitamin D_3_, with a safe upper limit for longer-term use of 15,000 IU per day [124,125,126,128]. In contrast, obese people require three to four times the above-mentioned dose to maintain a therapeutic blood 25(OH)D concentration to lower risks and mortality [40,41,388]. Nevertheless, for overall protection from all diseases (robust immunity against infections, cancer, autoimmunity, and heart disease) and to reduce all-cause mortality, the author recommends maintaining longer-term serum 25(OH)D concentrations above 50 ng/mL [44,50,54,55,56,129,130,131].

### 5.1. Varying 25(OH)D Levels Required for Preventing Different Diseases

**Figure 2:**
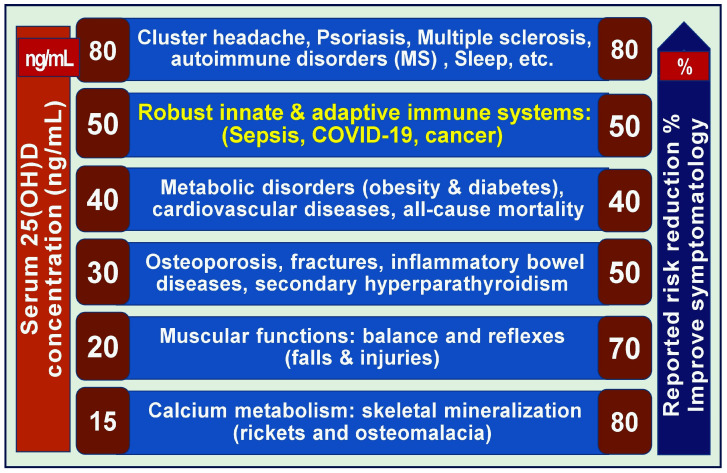
Different diseases (and tissues) require different steady-state serum 25(OH)D concentrations to achieve improvement. Minimum serum 25(OH)D concentrations are necessary to prevent or alleviate common diseases. Each column indicates the relationships between various disease states and the approximate minimum serum 25(OH)D concentrations required to improve different conditions (modified from Wimalawansa, S.J., 2023 [33]).

Numerous extra-skeletal disorders, including type 2 diabetes [390], metabolic syndrome [391], and all-cause mortality [392,393], demonstrate positive responses when maintaining serum 25(OH)D concentrations above 40 ng/mL [74,165,188,222]. This is functional but not at the minimal physiological level. It is necessary to maintain serum 25(OH)D concentrations above 40 ng/mL to achieve many benefits from vitamin D, particularly in cancer and autoimmunity [39,75,86], preferably above 50 ng/mL [33,49,55,394,395]. Figure 2 summarizes the varying steady-state serum 25(OH)D concentrations required to prevent or mitigate the effects of common diseases.

Estimates suggest that doubling the population’s serum 25(OH)D concentration could significantly reduce morbidities and decrease all-cause mortality [184,185]. Vitamin D’s role in promoting protein stabilization and reducing oxidation-related damage contributes to enhanced longevity and reduced healthcare costs. The current study shows that the most substantial health–economic benefits are observed when serum 25(OH)D concentrations are elevated and maintained above 40 ng/mL (100 nmol/L) [396]. A comprehensive review emphasizes the importance of providing balanced information on the costs and benefits and appropriate use of vitamin D supplements, as well as safe sun exposure, to the public, particularly for healthcare workers and policymakers [191,317,397,398].

### 5.2. The Role of Vitamin D-Binding—Protein in Cancer

Vitamin D-binding protein (VDBP), the carrier of vitamin D and its metabolites, plays a crucial role in maintaining these metabolites in circulation, transporting them to cells, and promoting health. Both vitamin D deficiency and VDBP status influence biological activities. Consequently, deficiency or abnormalities in VDBP unsurprisingly affect the function of vitamin D. For example, low levels of VDBP increase the onset as well as the aggressiveness of malignancy, as reported with breast, prostate, and colorectal [352,399,400].

Additionally, low VDBP levels are linked to certain cancers, including breast, colorectal [150], and prostate [352,399,400]. Although the estimated influences are weaker, studies are examining whether variants in genes that metabolize or transport vitamin D or its receptors (gene polymorphism) may impair the beneficial effects of vitamin D on cancer outcomes [322,401]. A specific form of VDBP, GC, has been suggested to improve cancer survival among both men and women in the US [323]. Nevertheless, neither of these conditions is modifiable.

Postmenopausal women who increased their vitamin D_3_ intake by 1100 IU reduced their relative cancer risk by 60 to 77% [89], providing strong support for vitamin D supplementation or safe sun exposure in adults. Additionally, declining 25(OH)D levels due to cirrhosis accelerate liver cancer progression and mortality [352]. Epidemiologic studies indicate that 25(OH)D levels below 20 ng/mL are associated with a 30 to 50% increased risk of these cancers and related deaths [172,350,351].

### 5.3. Adverse Effects of Vitamin

Vitamin D-related toxicity is rare in both children and adults, typically occurring after ingestion of doses exceeding 30,000 IU daily in non-obese persons for an extended period or acutely taking millions of units ingested by mistake [402,403,404]. Most adverse effects in adults result from substance misuse or accidental ingestion. The skin, liver, and kidneys have metabolic pathways to prevent excessive vitamin D production and activation of active metabolites—25(OH)D and 1,25(OH)_2_D, respectively. When overproduction occurs, catabolic pathways (mainly 24-hydroxylase) are activated, leading to the formation of inactive vitamin D metabolites [405]. Excessive sun exposure can increase the risk of skin damage [77], but it does not cause vitamin D toxicity [28,102].

Elevated 25(OH)D levels without hypercalcemia should prompt the discontinuation of vitamin D and investigation of the underlying cause. Unlike hypercalcemia, elevated 25(OH)D levels are not a medical emergency and, by themselves, are not considered vitamin D toxicity [49,54]. If excessive intake is suspected, vitamin D supplements, including multivitamins and vitamin A, should be paused temporarily. Once levels normalize, a lower dosage can be reintroduced. Most cases of vitamin D toxicity occur with serum concentrations exceeding 150 ng/mL, accompanied by hypercalcemia and hypercalciuria. Long-term supplementation of 10,000 IU/day or 50,000 weekly is considered safe [406]. Rarely, macrophage-driven autonomous production of 1,25(OH)_2_D can occur in granulomatous diseases, such as sarcoidosis and tuberculosis, leading to hypercalcemic syndrome [407,408].

## 6. Discussion

Vitamin D deficiency is common not only in individuals living at northern and southern latitudes but also in peri-equatorial regions. While UVB exposure stimulates vitamin D production in the skin, evidence suggests that sunlight offers broader health benefits beyond supplementation, with added advantages from natural dermal synthesis of vitamin D. Several reviews of epidemiological literature examine the relevant mechanisms and offer valuable insights [409,410]. Around one-third of clinical studies have focused on vitamin D’s role in disease prevention, while about one-fourth have reported inconsistent results or failed to show its benefits. Low serum 25(OH)D levels strongly correlate with higher cancer incidence and increased mortality [89,352,399,400], as well as worsening of other chronic diseases [20,34,35].

Despite gaps in evidence from well-designed RCTs, overall data consistently support the protective effects of vitamin D—especially when serum 25(OH)D concentrations exceed 40 ng/mL—including for the prevention of cancer and reduction of mortality [73]. This study highlights the need for well-designed, higher-quality clinical studies using proper, reproducible methodologies. The latter includes adequate statistical power, sufficient dose, and duration to evaluate hypotheses on vitamin D’s health effects. Notably, many studies fail to assess dose–response relationships between sunlight exposure, vitamin D intake, and serum 25(OH)D levels [1], with negative or inconclusive outcomes often stemming from such flawed designs. Ecological prospective studies and well-designed RCTs consistently support the pleiotropic benefits of vitamin D, including reduced cancer risk [100,101], attributed to its broad genomic and non-genomic effects [12].

The impact of vitamin D repletion on cancer incidence, mortality, and clinical outcomes is no longer hypothetical; the strongest available evidence supports its role in reducing cancer-related mortality. As illustrated in this systematic review, many recent clinical studies have addressed this issue and, having met scientific standards, strongly support the beneficial role of vitamin D in cancer, while others influenced by commercial interests fail to do so. This is unsurprising, as most negative studies have had inherently poor study designs. This review examined the role of vitamin D in cell growth and cancer, as well as its potential to reduce cancer risks and mortality. While vitamin D plays a significant role in cancer prevention and reducing mortality, it is one of many essential micro-nutrients vital for optimal health and survival.

Compared with mortality, cancer risk reduction has received less robust investigation in vitamin D intervention studies, partly because no clinical trials have been conducted yet using optimized micronutrient protocols. Notably, health extends beyond the absence of disease to include well-being, happiness, and productivity, and contributions to society (being productive), which require a health span and healthy life expectancy [411]. As the global population grows, more people are living longer, highlighting the need to prioritize the reduction of chronic diseases and improve the health span, especially cardiovascular diseases and cancer, as discussed in this SR. These are vital for promoting healthy aging and improving health span.

Recent data from epidemiological, cross-sectional, and longitudinal studies, with few exceptions [45], support the idea that maintaining serum 25(OH)D concentrations above 40 ng/mL, “ideally” between 50 and 80 ng/mL, reduces the incidence of many cancers, cancer-related mortality [222,412,413], and all-cause mortality [188,414]. However, progress in the vitamin D field is hindered by poorly designed RCTs, regardless of the study size or cost (e.g., the VITAL study [45]). 

Adequately powered studies with appropriate duration that test specific vitamin D-related hypotheses consistently report protective effects in individuals with vitamin D deficiency—serum 25(OH)D levels less than 20 ng/mL—and maintained serum 25(OH)D concentrations above 40 ng/mL [415]. Future vitamin D studies must prioritize large, prospective community-based ecological designs that specifically target predefined serum 25(OH)D concentrations, using vitamin D supplementation as the primary intervention—not as an add-on in pharmaceutical trials—to accurately assess risk reductions. Without meeting these criteria, outcome data will remain unreliable.

## 7. Conclusions

Vitamin D exerts broad systemic beneficial effects beyond skeletal health, significantly impacting immune regulation, gene expression, and disease prevention through autocrine, paracrine, and epigenetic mechanisms. This study underscores the importance of maintaining serum 25(OH)D concentrations between 50 and 80 ng/mL—well above current official recommendations—to realize these benefits fully. Non-obese individuals typically need 70–90 IU per kilogram of body weight daily to reach optimal levels. Obese individuals require several times higher doses (see Section 2). Achieving this requires a vitamin D dose about ten times higher than government recommendations of 400–600 IU/day for adults [and 20–30 ng/mL serum 25(OH)D levels], which are grossly outdated. Despite this, some recent clinical guidelines (e.g., American Endocrine Society, 2024) [416] still cite these inappropriately low intakes, harming the population. Adverse effects are extremely rare to occur below 150 ng/mL. 

Maintaining optimal serum vitamin D levels above 40 ng/mL reduces cancer incidence and mortality, along with multiple extra-skeletal benefits. Addressing widespread deficiency through safe, regular sun exposure and personalized supplementation offers a simple, cost-effective public health strategy. This approach lowers cancer and cardiovascular risks, eases chronic disease burdens, saves billions on healthcare costs, and protects lives. As shown in this review, therapeutic vitamin D status must be a core preventive measure in clinical guidelines and in routine clinical practice—not just a treatment.

## Figures and Tables

**Table 1:** PICOS elements and study design philosophies.

	PICOS Criteria	Conditions
1	**Participants**	Adults aged 18 to 80; de novo or diagnosis of a cancer
2	**Intervention**	Vitamin D, calcium and vitamin D, calcifediol, solar UVB exposure, Omega-3 fatty acids
3	**Comparison/control**	Retrospective, case report, observational, epidemiological, community-based/ecological, and randomized control studies, and longer-term follow-up studies related to cancer
4	**Outcome elements**	Morbidity, complications, and death; all-cause mortality. Relationship of serum 25(OH)D to the incidences and changes in cancer prevalence
5	**Study design philosophies**	Randomized controlled clinical trials, non-randomized controlled clinical trials, non-randomized non-controlled trials, and prospective and observational studies related to cancer are included.

**Table 2:** Vitamin D and its relation to various cancers and supporting references.

Cancer Type	References
All cancers	[94,147,187,189,190,192,193,194,195,205,206,207,208,209,210,211,212,213,214,215,216,217,218,219,220,221]
Breast cancer and survival	[150,151,163,189,190,192,193,194,215,216,217,218,219,220,222,223,224,225,226,227,228,229]
Colorectal cancers	[89,90,109,150,151,163,189,190,192,193,194,229,230,231,232,233,234]
Gastric cancers	[235,236,237,238]
Oral and nasopharyngeal carcinomas	[239,240]
Lung cancer	[241,242]
Pancreas and esophagus	[243,244,245]
Non-Hodgkins lymphoma	[119,246,247,248,249,250,251]
Melanoma	[1,103,252,253,254,255]
VDR polymorphisms	[211,256,257,258,259,260]
Cancer mortality	[39,41]
Relationship to living in higher latitudes	[89,107,189,190,261,262,263,264,265,266,267]
Relationship to serum 25(OH)D levels	[89,163,168,187,195,206,207,208,209,224,225,226,268,269,270,271]
	[70,167,168,272]
UVB/sun exposure and cancer reduction	[70,88,89,110,168,187,189,190,192,193,194,261,262,263,273,274,275,276,277,278,279,280,281,282,283,284]
Cancer metastasis	[285,286,287,288]

**Table 3:** Lifestyle modification factors have been shown to reduce the risk of cancer.

Recommendation	Reference	Recommendation	Reference
Maintaining a healthy weight at any age	[299]	Avoiding all forms of smoking and exposure to second-hand smoke	[299]
Engaging in regular physical activity	[308,309]	Breastfeeding infants	
Adopting a healthy diet, like the Mediterranean diet	[300]	Protecting the skin from excessive sun exposure	[299]
Avoidance or limiting alcohol intake to one drink per day for women and two for men	[299]	Being vaccinated against hepatitis B and HPV	[375]

## Supplementary Materials

The following supporting information can be reviewed at https://www.mdpi.com/article/10.3390/nu17142333/s1, File S1: PRISMA Checklist.

## Abbreviations

1,25(OH)_2_D	1,25-dihydroxyvitamin D
25(OH)D	25-hydroxy vitamin D
BMI	Body mass index
CKD	Chronic kidney disease
CVD	Cardiovascular disease
IU	International unit
kg BW	Kilogram, body weight
RCTs	Randomized controlled clinical trials
T2D	Type 2 diabetes mellitus
UV	Ultraviolet
VDR	Vitamin D receptor
