# Supplementary material for: Vitamin D’s Impact on Cancer Incidence and Mortality: A Systematic Review
Source: Nutrients. 2025 Jul 16;17(14):2333. doi: 10.3390/nu17142333 (PMC12298439; doi:10.3390/nu17142333)
Supplement: Supplementary file 1 [file nutrients-17-02333-s001.zip › nutrients-3609198-supplementary.pdf]

Vitamin D's Impact on Cancer Incidence and Mortality: A Systematic Review

Wimalawansa, S.J.

Appendix 1: PRISMA Checklist [1-3]

Section/topic	#	Checklist item
Title	1	Vitamin D's Impact on Cancer Incidence and Mortality: A Systematic Review
Structured summary	2	**Background**: Maintaining an adequate level of nutrients (focused on vitamin D) is crucial for physiological and pleiotropic actions, which include regulating cell growth and mechanisms, risks for cancer and incidence and deaths. Given its broader involvement in numerous physiological functions, it envisages that insufficient vitamin D levels may lead to pathological effects, including cancer development, the rate of spread, and deaths from cancer.<br><br>**Objectives**: This systematic review (SR) aimed to investigate the impact of vitamin D on cancer risks and deaths, following the guidelines outlined in the PRISMA (Preferred Reporting Items for Systematic Reviews and Meta-Analyses) framework [2,4]. SR assesses the evidence-based associations and potential causations between vitamin D levels/status and cancer.<br><br>**Data sources**: A thorough search was conducted across key databases, including PubMed, Medline, EMBASE, and the Cochrane Central Register of Controlled Trials, to gather evidence. The focus was on identifying original research and reviewing articles on vitamin D status and its broader implications in the general population, particularly concerning cancer.<br><br>**Methodology—*Protocol Development:*** A protocol was established to systematically track and categorize peer-reviewed publications, including observational studies, epidemiological studies, randomized trials, mechanistic studies, and hypothesis-generating research. The search used selected keywords such as vitamin D, 25-hydroxyvitamin D, 25(OH)D, calcitriol, and calcidiol, combined with terms related to cancer risk, incidence, mortality, and malignancy, drawn from MeSH and EMTREE thesauri.<br><br>This SR follows PRISMA and structured PICOS guidelines (Participants, Intervention, Comparison/Control, and Outcome), and Study design philosophies. We collected data from scientific publications that examined the relationship between vitamin D and cancer risks and mortality. Investigators critically analyzed and interpreted these data to assess vitamin D's impact on cancer incidence, the reduction of metastatic progression, and cancer mortality in humans. Besides, data were reviewed and independently evaluated by SJW and HB according to assigned tasks and resolved any disagreements through discussion.<br><br>**Study appraisal and synthesis methods—*Publication Evaluation:*** Relevant peer-reviewed publications were assessed per PRISMA guidelines. The evaluation focused on advancements in understanding the biology and physiology of vitamin D and its role in cancer prevention, progression, and outcomes. Studies were selected based on their scientific rigor and relevance to how vitamin D influences

		cancer risk, incidence, severity, and mortality.<br><br>**Results**: The data strongly support the relation of biology and physiology of vitamin D to cell proliferation and prevention of cancer in humans. These are discussed in detail within the manuscript.<br><br>**Conclusions and implications of key findings**: The data extracted from this review strongly support that maintaining sustained physiological serum 25(OH)D concentrations significantly reduces the risk and mortality of most cancer types. Overall, levels above 40 ng/mL—ideally within the 40 to 80 ng/mL range—are necessary for optimal physiological and metabolic functions [5,6], showing both direct and indirect associations with cell proliferation and cancer progression. Circulating 25(OH)D concentrations above 50 ng/mL were consistently associated with improved clinical outcomes.
Rationale	3	**The rationale for the study:**<br>While the effects of vitamin D on skeletal tissues are well-established, its biological and physiological roles in non-skeletal tissues remain less clearly understood. Over the past two decades, thousands of studies have reported both positive and negative effects of vitamin D on extraskeletal systems, including its influence on cancer. This study was designed to assess those relationships.<br><br>Vitamin D plays a vital role in regulating cell proliferation, differentiation, apoptosis, cytokine production, growth factors, hormones, and cellular signaling—functions that are critical in preventing cancer development and metastatic progression.<br><br>Although the precise mechanisms by which calcitriol exerts its effects on extraskeletal tissues are not yet fully elucidated, numerous studies in recent years have shed light on these interactions. This systematic review aimed to evaluate the role of vitamin D in cancer, particularly regarding its impact on risk reduction, incidence, and mortality.
Objectives	4	Most of what we know about vitamin D's role in cancer comes from retrospective analyses, case reports, and epidemiological studies [7-9]. Although researchers have conducted thousands of studies on various cancer types and aspects related to vitamin D, relatively few have carried out randomized controlled trials (RCTs). Still, the overwhelming majority of published reports show that vitamin D lowers cancer risk and reduces the severity and metastatic spread of the disease.<br><br>Most studies emphasize the importance of maintaining serum 25(OH)D concentrations above 40 ng/mL to achieve vitamin D's metabolic and anti-inflammatory benefits, including reduced cancer incidence and mortality. However, findings from several recent large-scale RCTs—many of which were industry-sponsored and poorly designed—have yielded conflicting and misleading results. These discrepancies are primarily due to flawed methodologies and execution in those trials.<br><br>To advance the field, there is a critical need for unbiased, rigorously designed, and well-conducted RCTs. In parallel, large-scale, long-term observational studies are also essential to better understand the physiological mechanisms by which vitamin D reduces cancer risk and mortality.

Eligibility criteria	5	Specify study characteristics (e.g., PICOS, length of follow-up) and report characteristics (e.g., years considered, language, publication status) used as criteria for eligibility, giving rationale.
Information sources	7	We systematically evaluated evidence on vitamin D's biology and physiology, focusing on its role in cell proliferation and human cancer. We critically appraised randomized controlled trials and observational studies [2] and synthesized our findings into narrative conclusions. We omitted any publications that did not address the core topic.<br><br>Information sources for this review included PubMed, Medline, EMBASE, and the Cochrane Central Register of Controlled Trials. The search employed keywords such as vitamin D, 25-hydroxyvitamin D, 25(OH)D, calcitriol, and calcidiol. The search strategy adhered to PRISMA guidelines and the PICOS framework, ensuring a thorough, systematic approach to identifying pertinent scientific literature [2].
Search	8	To identify and monitor relevant research areas and publications systematically, a protocol was developed. The review encompassed a range of study types, including observational studies, epidemiological studies, randomized controlled trials, and mechanistic or hypothesis-generating research. This broad approach ensured comprehensive coverage of evidence on the role of vitamin D in cell proliferation and cancer.<br><br>An initial search of public databases using the terms "vitamin D" and "cancer" yielded about 4,000 peer-reviewed publications. The search strategy included controlled terms such as "vitamin D" (encompassing cholecalciferol, 25(OH)D, and 25-hydroxycholecalciferol), "25-hydroxyvitamin D," "25(OH)D," "calcitriol," and "calcidiol," selected from Medical Subject Headings (MeSH), and combined with terms like "cancer" and "malignancy." These terms were used in various combinations to narrow the number of relevant manuscripts. A secondary search extended the coverage to March 2025 and included the term "mortality." The total results are summarized in the PRISMA diagram (Figure 1).
Study selection	9	Articles were selected based on predefined criteria to ensure their relevance to the topic under consideration. The chosen studies underwent thorough assessment and analysis within broader conceptual frameworks. The inclusion criteria encompassed articles published in English from January 1991 to March 2023. Specific data are detailed in the manuscript. The full texts of 421 manuscripts were meticulously reviewed and incorporated into this systematic review using EndNote 21.4 (Thomson Reuters), as illustrated in Figure 1.
Data collection process	10	The authors followed guidance from the Equator Network (www.equator-network.org/), the PRISMA statement, and the PRISMA-P checklist [2,10] for assessing the quality of the literature.
Databases and items	11	The PubMed, Medline, and EMBASE databases and the Cochrane Central Register of Controlled Trials were searched for original and review articles on vitamin D status and related broader aspects in the general population.
Risk of bias in individual	12	Not applicable

studies		
Summary measures	13	Not applicable
Synthesis of results	14	Assessments were conducted by considering the rationale and objectives of each study, eligibility criteria defined by the evidence-based PICOS process, data collection methods, search strategy, quality of outcomes, and potential meta-bias within the data. The strength of the evidence was evaluated with respect to the biology and physiology of vitamin D, focusing particularly on its relevance to cell proliferation, cancer risk, and cancer mortality in humans. Synthesized results are presented as narrative conclusions, incorporating a critical appraisal of evidence from RCTs as well as ecological and observational studies.
Risk of bias across studies	15	Not applicable
Additional analyses	16	Not applicable
Results		Results are presented in the main body of the text narratively.
Study characteristic s	17	For each study, present characteristics for which data were extracted (e.g., study size, PICOS, follow-up period), tabulated, and provide the citations.
Risk of bias within studies	18	Present data on the risk of bias of each study and, if available, any outcome level assessment.
Results of individual studies	19	In evaluating outcomes, both benefits and harms were considered in each study. This involved examining (a) summary data to present information for each intervention group and (b) effect estimates along with confidence intervals, preferably illustrated with a forest plot.
Synthesis of results	20	The results of each meta-analysis, including confidence intervals and consistency measures, were taken into account when interpreting data and presented to provide a comprehensive overview of the findings.
Risk of bias across studies	21	The results of any assessment of the risk of bias across studies, as outlined, were presented to evaluate the quality and reliability of the included studies.
Additional analysis	22	Results/data were analyzed for sensitivity and bias via subgroup analyses and meta-regression.
**DISCUSSION**	23	Certain areas, such as specific cancer types like prostate and pancreatic cancers, lack definitive evidence from well-designed, adequately powered studies; however, the overall data support significant protective effects of vitamin D in humans. Maintaining serum 25(OH)D concentrations above 40 ng/mL (within a range of 40 to 80 ng/mL) is beneficial not only for cancer prevention but also for many other common disorders [5,11]; a threshold of over 50 ng/mL is better correlated with clinical outcomes and thus, recommended for overcoming

		infections, autoimmunity [12], chronic diseases [13], cancer [14], and to reduce all-cause mortality [15].<br>The current study underscores the imperative for well-designed, high-quality clinical studies and proper clinical protocols, emphasizing the importance of avoiding flawed study designs. This includes ensuring adequate statistical power, sufficient sample size, and appropriate study duration to effectively validate hypotheses regarding the health consequences associated with vitamin D.<br>In addition, this SR highlights deficiencies in many previously published studies (e.g., recent large RCTs), as they fail to assess baseline serum 25(OH)D concentrations, dose-response relationships, and the links between sunlight exposure or vitamin D intake (OTC) and resulting circulatory 25(OH)D levels with health outcomes. Most reported negative or inconclusive findings stem from studies with flawed designs, as noted in this SR. Nevertheless, nearly all well-designed RCTs consistently support the pleiotropic benefits of vitamin D, including reduced risks of certain cancers—an alignment that reflects its broader genomic and nongenomic effects [16,17].<br>The SR focused on vitamin D's role in cell growth and its implications for cancer prevention. Proper functioning of the vitamin D physiological system is crucial for preventing diseases, including cancer. Despite physiologic concentrations of calcitriol, inadequate vitamin D status—an essential, cost-effective measure for better health and survival—can increase disease risk and reduce longevity.
CONCLUSIONS	24	The progress in vitamin D research is impeded by flawed designs in recent large RCTs—some of which appear deliberately set up to fail—regardless of participant numbers or study cost. Adequately powered studies, with appropriate duration and dosing to test specific vitamin D–related hypotheses, recruit participants with deficiency (serum 25(OH)D < 20 ng/mL) and achieve and maintain target serum 25(OH)D concentrations; these studies consistently report protective effects of vitamin D in common disorders, including cancer.<br>Future trials should prioritize predefined serum 25(OH)D concentrations rather than the administered vitamin D dose. Vitamin D supplementation should be the primary intervention, focusing on reductions in cancer incidence and mortality as endpoints. Without adherence to these criteria, outcome data lack reliability.
Funding and conflicts of interest	25	The authors declare no conflicts of interest and did not receive any funding or professional writing assistance for this review.

References:

1. Moher, D.; Liberati, A.; Tetzlaff, J.; Altman, D.G.; Group, P. Preferred reporting items for systematic reviews and meta-analyses: the PRISMA statement. *PLoS medicine* **2009**, *6*, e1000097, doi:10.1371/journal.pmed.1000097.
2. Shamseer, L.; Moher, D.; Clarke, M.; Ghersi, D.; Liberati, A.; Petticrew, M.; Shekelle, P.; Stewart, L.A.; Group, P.-P. Preferred reporting items for systematic review and meta-analysis protocols (PRISMA-P) 2015: elaboration and explanation. *BMJ* **2015**, *350*, g7647, doi:10.1136/bmj.g7647.
3. Welch, V.; Petticrew, M.; Tugwell, P.; Moher, D.; O'Neill, J.; Waters, E.; White, H.; group, P.R.-E.B. PRISMA-Equity 2012 extension: reporting guidelines for systematic reviews with a focus on health equity. *PLoS Med* **2012**, *9*, e1001333, doi:10.1371/journal.pmed.1001333.

4. Page, M.J.; McKenzie, J.E.; Bossuyt, P.M.; Boutron, I.; Hoffmann, T.C.; Mulrow, C.D.; Shamseer, L.; Tetzlaff, J.M.; Akl, E.A.; Brennan, S.E.; et al. The PRISMA 2020 statement: an updated guideline for reporting systematic reviews. *BMJ* **2021**, *372*, n71, doi:10.1136/bmj.n71.
5. Wimalawansa, S. Overcoming infections including COVID-19, by maintaining circulating 25(OH)D concentrations above 50 ng/mL. *Pathology & Lab. Medicine Int.* **2022**, *14*, 37–60, doi:10.2147/PLMI.S373617.
6. Wimalawansa, S.J. Physiological Basis for Using Vitamin D to Improve Health. *Biomedicines* **2023**, *11*, doi:10.3390/biomedicines11061542.
7. Armas, L.A.; Hollis, B.W.; Heaney, R.P. Vitamin D2 is much less effective than vitamin D3 in humans. *J Clin Endocrinol Metab* **2004**, *89*, 5387-5391, doi:10.1210/jc.2004-0360.
8. Wimalawansa, S.J. Vitamin D in the new millennium. *Curr Osteoporos Rep* **2012**, *10*, 4-15, doi:10.1007/s11914-011-0094-8.
9. Wimalawansa, S.J. Non-musculoskeletal benefits of vitamin D. *J Steroid Biochem Mol Biol* **2018**, *175*, 60-81, doi:10.1016/j.jsbmb.2016.09.016.
10. Moher, D.; Shamseer, L.; Clarke, M.; Ghersi, D.; Liberati, A.; Petticrew, M.; Shekelle, P.; Stewart, L.A.; Group, P.-P. Preferred reporting items for systematic review and meta-analysis protocols (PRISMA-P) 2015 statement. *Syst Rev* **2015**, *4*, 1, doi:10.1186/2046-4053-4-1.
11. Wimalawansa, S.J. Rapidly Increasing Serum 25(OH)D Boosts the Immune System, against Infections-Sepsis and COVID-19. *Nutrients* **2022**, *14*, doi:10.3390/nu14142997.
12. Wimalawansa, S.J. Infections and autoimmunity-The immune system and vitamin D: A systematic review. *Nutrients* **2023**, *15*, doi:10.3390/nu15173842.
13. Wimalawansa, S.J. Controlling chronic diseases and acute Infections with vitamin D sufficiency. *Nutrients* **2023**, *15*, doi:10.3390/nu15163623.
14. Grant, W., Wimalawansa SJ, Pludowski P, Cheng R. Vitamin D: Evidence-based health benefits and recommendations for population guidelines. *Nutrients* **2025**, *17*, 277, doi:10.3390/nu17020277.
15. Wimalawansa, S.J. Physiology of Vitamin D-Focusing on Disease Prevention. *Nutrients* **2024**, *16*, doi:10.3390/nu16111666.
16. Verstuyf, A.; Carmeliet, G.; Bouillon, R.; Mathieu, C. Vitamin D: a pleiotropic hormone. *Kidney Int* **2010**, *78*, 140-145, doi:10.1038/ki.2010.17.
17. Cancela, L.; Nemere, I.; Norman, A.W. 1 alpha,25(OH)2 vitamin D3: a steroid hormone capable of producing pleiotropic receptor-mediated biological responses by both genomic and nongenomic mechanisms. *J Steroid Biochem* **1988**, *30*, 33-39, doi:10.1016/0022-4731(88)90073-8.
